# ^13^C dicarboxylic acid signatures indicate temporal shifts in catchment sediment sources in response to extreme winter rainfall

**DOI:** 10.1007/s10311-023-01684-1

**Published:** 2024-01-10

**Authors:** Hari Ram Upadhayay, Adrian Joynes, Adrian L. Collins

**Affiliations:** Net Zero and Resilient Farming, Rothamsted Research, North Wyke, Okehampton, EX20 2SB UK

**Keywords:** Sediment fingerprinting, Land use, Suberin, Bayesian unmixing model

## Abstract

Rainfall and land-use interactions drive temporal shifts in suspended sediment sources, yet the magnitude of such changes remains poorly understood due to the lack of land-use specific source tracers. We investigated α,ω-dicarboxylic fatty acid root-specific biomarkers, as diagnostic tracers for apportioning sources of time-integrated suspended sediment samples collected from a grassland dominated agricultural catchment in the southwest of England during the wet winter period. Applying fatty acids-specific stable carbon isotope analysis and a Bayesian isotope mixing model, we show that stream banks contributed most of the sediment in the early winter, i.e. October–December, while winter cereal-dominated arable land contributed more than half of the sediment during the late winter, i.e. January–March. The dominant sediment source shifted in conjunction with a period of prolonged consecutive rainfall days in the later period suggesting that intervention required to mitigate soil erosion and sediment delivery should adapt to changing rainfall patterns. Our novel findings demonstrate that isotopic signatures of α,ω-dicarboxylic fatty acids are promising tracers for understanding the resistance of agricultural soils to water erosion and quantifying the interactive effects of extreme rainfall and land use on catchment sediment source dynamics.

**Supplementary Information:**

The online version contains supplementary material available at 10.1007/s10311-023-01684-1.

## Introduction

Human activities in tandem with extreme rainfall have accelerated erosion-generating excess sediment inputs into aquatic ecosystems (Foucher et al. 2021). Assembling reliable information on the relative contributions of various sources to sediment is complicated due to spatio-temporal variability in erosion processes and the need to identify land use-specific source tracers. Biotracer-based especially fatty acids and alkanes sediment source fingerprinting has shown promising results due to the strong linkage between biomarker isotopic signatures and land use (Upadhayay et al. 2022). However, these biotracers achieved limited success in discriminating functionally similar land use-based sources (Vale et al. 2022; Upadhayay et al. 2020), especially when attempting to document temporal variability in source contributions (Hirave et al. 2021). Best management for sediment-related problems can only be targeted successfully when the spatio-temporal dynamics of sediment source contributions are documented accurately.

Studying root-derived biomarkers in soil and sediment can help us to better understand land use (Jansen and Wiesenberg 2017) and responses to extreme rainfall. Suberin is one of the important biopolymers in roots consisting of alternating layers of aliphatic (fatty acids, alcohols, ω-hydroxy acids, α,ω-diacids) and aromatic compounds (Harman-Ware et al. 2021). The aliphatic portion comprises light lamellae structured by glycerol-α,ω-diacid-glycerol but also containing α,ω-hydroxyacid-glycerol unit with a characteristic chain length of 22 or 24 carbon atoms (Serra and Geldner 2022). Although long-chain (C_20_–C_32_) α,ω-dicarboxylic fatty acids (diFAs) comprise about 0.3% of total soil lipids (Holtvoeth et al. 2016), they are a major component of suberin (Serra and Geldner 2022). Given their chain length-dependent hydrophobicity, the degradation of diFAs in soil decreases with increasing chain length (Kashi et al. 2023). Therefore, these diFAs are considered very robust indicators of root inputs to soils (Mendez-Millan et al. 2011).

Dicarboxylic fatty acids and their associated isotopes can be utilised as source tracers to investigate transfers of land use-based sediment to aquatic systems due to their stability during transport in river systems (Feng and Simpson 2008). Recently, Pondell and Canuel (2020) reported that diFAs in sediment can differ in response to floods or dam construction. Despite their ubiquitous presence in soil and sediment, diFAs remain poorly investigated biomarkers in terms of their distribution in sediment sources spanning the land-use spectrum. The suitability of diFA-associated isotope values as fingerprints for quantifying sediment source contributions to rivers has not been tested at catchment scale. It is thus very timely to explore diFA properties and establish whether these biomarkers are robust tracers for sediment source contributions in agricultural landscapes prone to sediment-related problems.

The aim of this research was to explore how diFAs might be used to provide information on temporal patterns in catchment sediment source contributions. We sampled winter 2019–2020 sediment from a grassland-dominated agricultural catchment in the UK. The winter of 2019–2020 was the 5th wettest on record in the UK meaning that it was a suitable study period for sediment source dynamics. The specific objectives were to: (1) assess diFA content and associated ^13^C signatures in soils from arable land, pastureland, woodland and stream banks for potential sediment source discrimination, and (2) estimate the relative contributions of the sediment sources to sediment sampled during winter 2019–2020. This study complements earlier work using more conventional biomarkers (Upadhayay et al. 2022).

## Materials and methods

### Study catchment

The study was undertaken within a grassland-dominated lowland agricultural catchment (4.5 km^2^) in southwest England (Fig. S1a). See Upadhayay et al. (2022) for details. Briefly, the catchment is dominated by pasture (62%), followed by arable land (23%) and woodland (15%). High-risk crops for erosion and sediment generation are grown on the arable land, including winter wheat and barley, field beans and maize. A ryegrass-clover mix dominates the pasture. Woodland is mostly concentrated in the riparian zone (Fig. S1a). Long-term mean winter, i.e. October–March rai*n*fall is approximately 661 mm (1981–2010).

### Surface soil and sediment sampling

Composite soil samples were collected to characterise each land use; arable (*n* = 17), pasture (*n* = 19) and woodland (*n* = 6). For each sampling point, approximately 10 topsoil, i.e. 2 cm sub-samples were collected randomly using a 5 cm diameter corer and composited. Samples were also taken from eroding stream bank profiles (*n* = 11). Soil samples were freeze dried and sieved through a 63 µm sieve based on the sediment particle size distribution (Upadhayay et al. 2022).

Time-integrated suspended sediment samples were collected from the catchment outlet (Fig. S1a) from October 2019 to April 2020, i.e. the winter of 2019–2020 which received about 800 mm of rainfall (Fig. S1b). Sediment samples were retrieved at the end of December 2019 (hereafter referred to as early winter; EW) and early April 2020 (hereafter referred to as late winter; LW). Sediment samples were freeze dried.

### Extraction of dicarboxylic acids and determination of ^13^C signatures

Ester-bound dicarboxylic fatty acids were extracted from dried samples by sequential chemical extraction, i.e. solvent extraction followed by alkaline hydrolysis (Upadhayay et al. 2022). The individual dicarboxylic acids were quantified and their ^13^C signatures determined using GC-MS and GC-c-IRMS, respectively. The carbon isotopic results of diFA were expressed as natural abundance (δ) in parts per mil (‰) compared to international standards, i.e. Vienna Peedee Belemnite (VPDB). For details see Text S1.

### Statistical analysis and source apportionment modelling

The homologous series of saturated dicarboxylic fatty acids with carbon numbers ranging from C_16_ to C_28_ were considered for statistical analysis. The δ^13^C values of long-chain diFAs (C_18_–C_26_) were used as sediment tracers in a concentration-dependent Bayesian mixing model (MixSIAR). Median and 90% Bayesian confidence intervals of the posterior source contributions were generated. For details see Text S2.

## Results and discussion

### Variation in dicarboxylic fatty acid content and associated ^13^C signatures across sediment sources

**Fig. 1:**
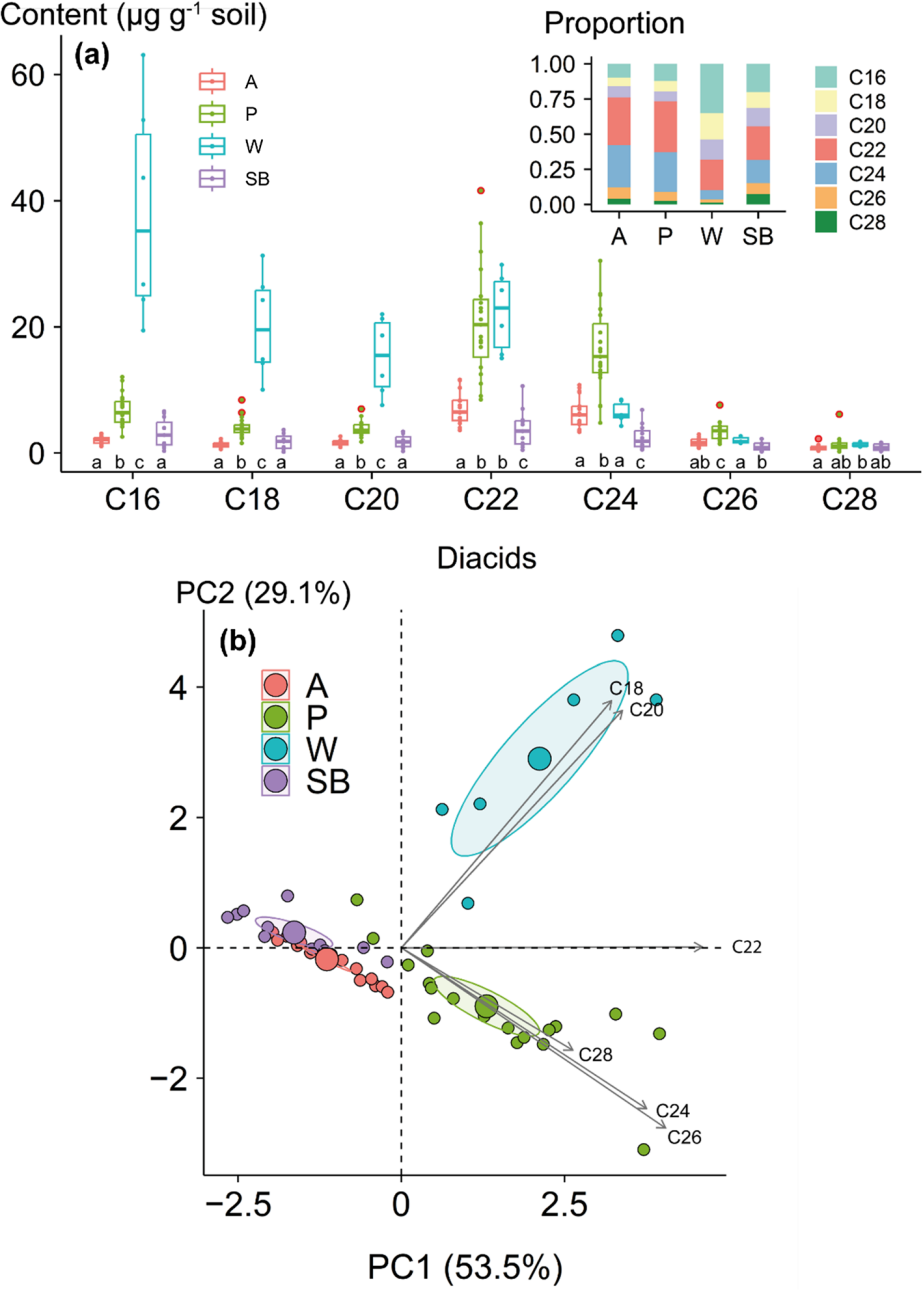
**a** Variation of α,ω-dicarboxylic acid (diFA) content across the sediment sources (A = arable, P = pasture, W = woodland, SB = stream banks); **b** and results of principal component analysis using diFA contents. Different lowercase letters indicate significant differences between diFA contents. Red dots represent outliers. The ellipses represent 95% confidence intervals for the corresponding means

The total diFA (C_16_–C_28_) content was highest in the woodland soil (105.9 ± 37.3 µg/g soil) followed by the pasture (56.9 ± 19.7 µg/g soil), arable (20.8 ± 6.9 µg/g soil) and stream bank (15.14 ± 10.4 µg/g soil) soils. The relative abundance of two dominant diFAs, i.e. C_22_ and C_24_ were similar in arable and pasture soils and contributed around 60% of the total diFA content (Fig. 1a). The content of C_22_ and C_24_ in pasture surface soil was about three times higher compared to arable surface soil, suggesting pronounced diFAs degradation with intensive arable land use and/or higher root carbon inputs in pastureland (McNally et al. 2015) The similar diFA contents and compositions in the arable and stream bank soils (Fig. 1b) also reflected lower root inputs and degradation of diFAs in arable soils facilitated by tillage and agricultural inputs (e.g. fertilizer and lime). Continuous tillage can result in aggregate breakdown and enhancement of suberin component availability for degradation. The diFA contents observed in this study were comparable to those reported by previous studies (Pisani et al. 2016). In the woodland soils, C_16_ and C_18_ diFAs contributed about 50% of the total diFAs (Fig. 1a), which can be explained by their higher contents in tree roots and accumulation over time (Spielvogel et al. 2014).

**Fig. 2:**
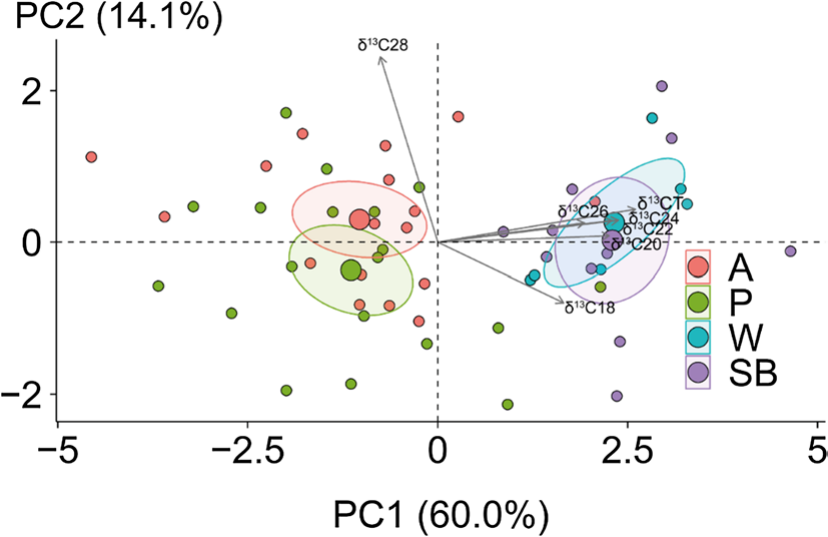
Principal component analysis of δ^13^C values (‰) of α,ω-dicarboxylic fatty acids in the sediment sources (A = arable, P = pasture, W = woodland, SB = stream banks) indicates A and SB are similar. Ellipses represents 95% confidence intervals of the corresponding means

The carbon isotopic signature of soil diFAs ranged from − 41.1 to − 29.5‰ in arable, from − 39.5 to − 32.4‰ in pasture, from − 35.9 to − 30.9‰ in woodland and from − 40.1 to − 29.3‰ in stream banks. The measured ^13^C signatures of diFAs are in agreement with C3 biosynthetic pathways and similar to those diFAs observed in wheat roots (Mendez-Millan et al. 2011), grass (*Dactylis glomerata, Festuca arundinacea* and *Lolium perenne*) roots and associated soils (Armas-Herrera et al. 2016). The similar δ^13^C values of diFAs for arable and stream bank samples (Fig. 2, Table S1) suggested that these biomarkers are preserved in soil without significant alteration of ^13^C signatures. The δ^13^C values of C_28_ further indicate that long-chain diFAs are very stable in the soil due to the high energy requirement for uptake by microorganisms (Kashi et al. 2023).

### Temporal variations in sediment dicarboxylic fatty acid signatures linked to sediment source contributions

**Fig. 3:**
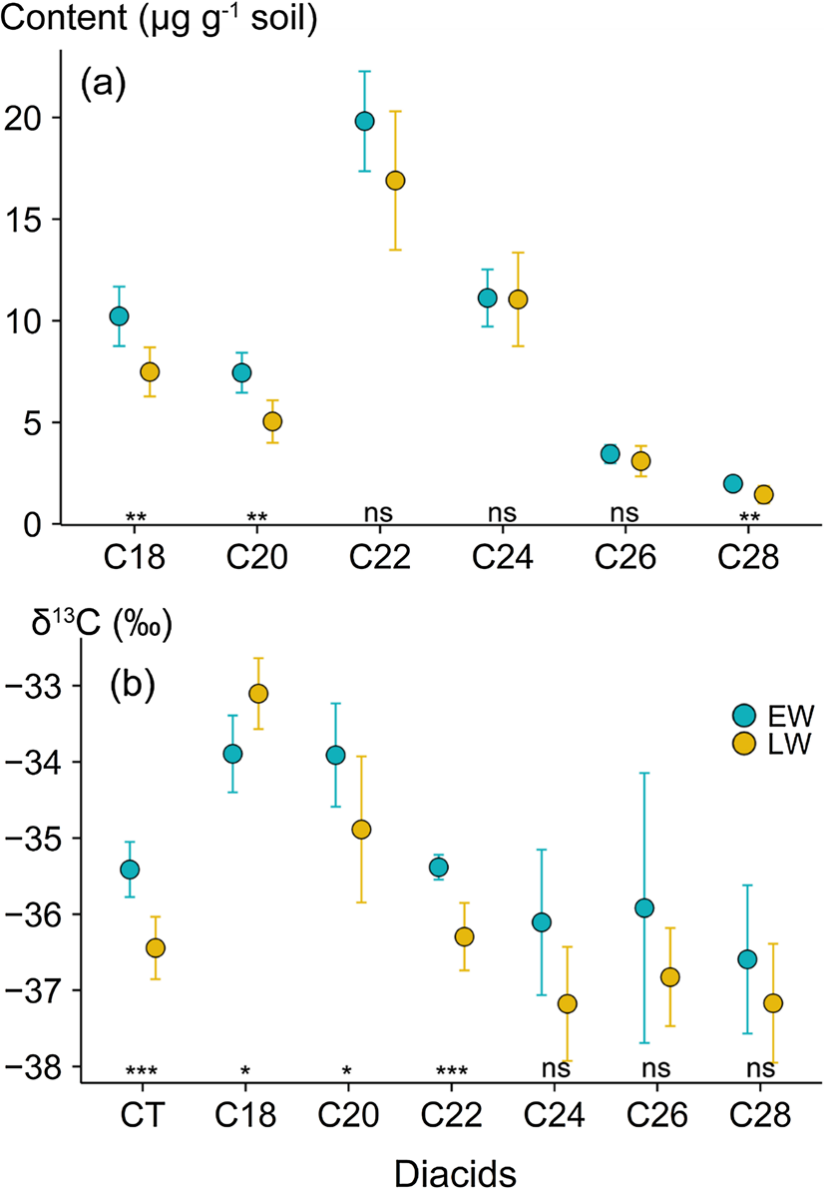
Statistical differences (*** < 0.001, ** < 0.01, * < 0.05, ns = not significant) of α,ω-dicarboxylic fatty acids; **a** content; **b** corresponding δ^13^C values between early winter (EW) and late winter (LW) sediment. CT indicates the content weighted δ^13^C values of long-chain diFAs (C_20_–C_28_)

**Fig. 4:**
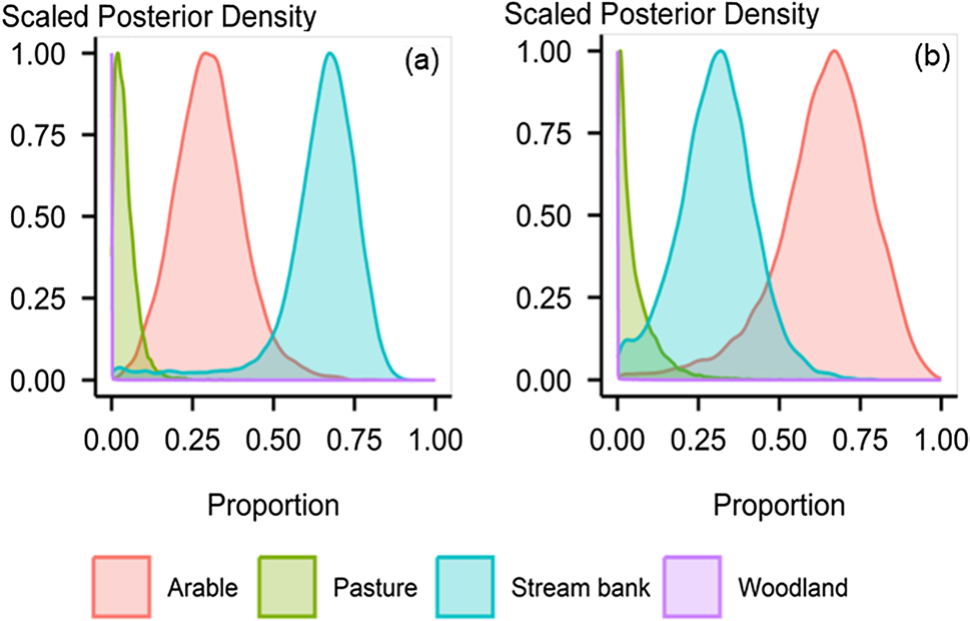
Estimated sediment source contributions; **a** early winter (EW); **b** late winter (LW) using MixSIAR with prior information. Results show switches in source contributions

The diFA content and corresponding ^13^C signatures varied significantly between EW and LW sediment (Fig. 3). The observed temporal shift in the diFA content and the associated ^13^C signal in sediment can reflect changes in the sediment sources and transport pathways over time. The estimated source contributions suggested that, during EW, stream banks were the dominant source with a median contribution of 66% (90% credible interval ranging from 44 to 79%), followed by arable land (median 30%; CI 14–46%) (Fig. 4a). In contrast, the arable land was dominant during LW, with a median contribution of 65% (90% CI 35–85%) (Fig. 4b). The significant temporal shift in source contributions suggested that diFA tracers can reveal significant responses to the impact of extended rainfall (Fig. S3) on high-risk arable land, confirming that these biotracers are robust indicators of soil organic matter sources in sediment (Pondell and Canuel 2020).

The proportion of sediment contributed from stream banks in this study is within the range, i.e. 4–84% reported for UK streams and rivers (Abbas et al. 2023). High stream bank contributions during EW may be explained by livestock access to the channel. Livestock can degrade stream banks through dislodging trampled soil, which loses cohesive strength following saturation (Terry et al. 2014). The higher arable land contribution during LW was expected due to its spatial distribution on steep slopes (Fig. S1), exposure of arable bare soils during the winter and high antecedent soil moisture (Fig. S1b) combined with prolonged rainfall (Fig. S3). Aggregate stability decreases when soil is completely saturated (Moragoda et al. 2022) reducing resistance to erosion. Consequently, saturation-excess overland flow and rill erosion can enhance runoff and sediment delivery. In a recent field scale study, the authors reported around a 77% higher sediment flux from arable land during LW compared to EW in the study catchment (Upadhayay et al. 2022).

The diFAs used in this study are very robust indicators of root inputs in the soil, which are protected in soil microaggregates (Genest et al. 2014) and can travel long distances. Source contribution insensitivity to prior information (Fig. S3) demonstrated that diFAs are very robust tracers for apportioning catchment sediment sources. This assertion is further supported by the reported absence of diFAs in aquatic plants (Pondell and Canuel 2022) and the presence of very low contents in the above ground tissues of terrestrial vegetation (Otto and Simpson 2006). Therefore, any potential uncertainties in the estimated source contributions introduced by corrupted tracer values due to riparian vegetation and algae accrual in the stream sediment are negated by using diFAs as tracers. The robust evidence for switches in sediment sources during prolonged wet periods provided this study should encourage farmers to implement sediment mitigation to adapt to changing rainfall patterns both now and in the future.

## Conclusion

Long-chain dicarboxylic fatty acid content and corresponding ^13^C signatures varied in time-integrated suspended sediment samples. Variation was associated with a significant shift in the contribution of arable land to LW sediment. Prolonged heavy rainfall reduced the resistance of arable land to erosion and accelerated sediment delivery during the LW period. The novel results demonstrated that dicarboxylic fatty acids are responsive to changes in source contributions and thereby offer substantial promise for use as biotracers. Temporal sediment source apportionment can inform soil conservation and sediment management.

### Supplementary Information

Below is the link to the electronic supplementary material.

Supplementary file1 (DOCX 486 KB)
