# Supplementary material for: 13C dicarboxylic acid signatures indicate temporal shifts in catchment sediment sources in response to extreme winter rainfall
Source: Environ Chem Lett. 2024 Jan 10;22(2):499–504. doi: 10.1007/s10311-023-01684-1 (PMC10907443; doi:10.1007/s10311-023-01684-1)
Supplement: Supplementary file 1 — Supplementary file1 (DOCX 486 KB) [file 10311_2023_1684_MOESM1_ESM.docx]

Supplementary material

^13^C dicarboxylic acid signatures indicate temporal shifts in catchment sediment sources in response to extreme winter rainfall

Hari Ram Upadhayay^*^, Adrian Joynes, Adrian L. Collins

*Net Zero and Resilient Farming, Rothamsted Research, North Wyke, Okehampton, EX20 2SB UK.*

*Corresponding author: [hari.upadhayay@rothamsted.ac.uk](mailto:hari.upadhayay@rothamsted.ac.uk)

**Text S1: Dicarboxylic fatty acids extraction, quantification and ^13^C signature measurement**

Briefly, 1 g solvent extracted soil and sediment residues were subjected to alkaline hydrolysis at 100 °C for two hours in a glass tube with 3 ml of 0.5 methanolic potassium hydroxide (9 methanol : 1 H_2_O). The suspension was centrifuged after cooling and the supernatant solution was transferred into a glass vial. The residue was sonicated for 10 minutes with a dichloromethane: methanol (9:1, v:v) solution, centrifuged and extracts combined. The resulting solution pH was adjusted to <2 and lipids were extracted with dichloromethane (2 mL, three times). The volume of lipid solution was evaporated to dryness on a dry-block heater (40 °C) fitted with a sampler concentrator blowing N_2_ into the tube. Dicarboxylic acids were methylated with 14 % BF_3_ in methanol at 70 °C for 30 min.

The details of the GC-MS and GC-c-IRMS set up parameters are described below.

| Parameters | Agilent 6890N/5973N GC-MS | GC-c-IRMS |
| --- | --- | --- |
| Autosampler |  | PAL AS200 autosampler (CTC analytics, Switzerland) |
| Injector | Splitless | Splitless |
| Injection temperature (°C) | 300 | 300 |
| Oven program | Held at 50 °C for 2 min, then ramp at 10 °C min^-1^ to 320 °C and held for 11 min | For alkanes: Held at 40 ^o^C for 1 min, then ramped to 130 ^o^C at 20 ^o^C min^-1^, then 4 ^o^C min^-1^ to 300 ^o^C and held for 10 min  For fatty acids: Held at 50 °C for 2 min, then ramp at 10 °C min^-1^ to 320 °C and held for 11 min |
| GC-column | Agilent HP-5MS capillary column (30 m x 250 μm x 0.25 μm) | Varian CP-SIL 5CB (50 m x 320 µm x 0.12 µm) (Varian Inc. California, US) |
| Carrier gas and flow | Helium at 1ml min^-1^ | Helium at 1.4 ml min^-1^ |

Dicarboxylic fatty acids were identified by retention times and their characteristic mass spectra. The DiFAs content was quantified against the C_19_ FA internal standard. The diFA ^13^C values were determined using a Finnigan mat 6890 GC coupled to a Finnigan Mat Delta Plus IRMS via a Combustion III interface. The carbon isotopic results of diFA were expressed as natural abundance (δ) in parts per mil (‰) compared to international standards i.e., Vienna Pe De Belemnite (VPDB). The methyl group correction was applied to all diFAs δ^13^C values using the mass-balance procedure described by Kawamura andWatanabe (2004).

**Text S2 Statistical analysis and MixSIAR formulation**

Shapiro -Wilks and Levene’s tests were used to evaluate any deviation of saturated diFAs (carbon numbers ranging from C_16_ to C_28_) content and δ^13^C values from normal distributions and the homogeneity of variance, respectively. Differences in diFAs content and associated δ^13^C values among sediment sources were compared using the non-parametric Kruskal-Wallis test and one-way ANOVA followed by a Tukey HSD test at a significance level of 0.05, respectively. Principal component analysis (PCA) was applied to compare the distribution of diFAs content and δ^13^C values in the sampled sediment sources.

In this study, the ^13^C signature of long-chain dicarboxylic fatty acids (C_18_–C_26_) were used as tracers for source apportionment of the time-integrated stream suspended sediment. In order to account for periodic differences in soil erosion and sediment delivery in the winter period, the winter period i.e., early winter (EW) and late winter (LW) was used as a covariate incorporated via a fixed effect in MixSIAR. MixSIAR was formulated with and without prior information on each individual source’s contribution to the sampled suspended sediment. The estimated median sediment load from the individual sources to the catchment outlet (i.e., 80, 56, 40, 1 t year^-1^ for arable, pasture, stream banks and woodland, respectively; Upadhayay et al. (2022)) was used as prior information. Sediment source contributions were estimated using the Markov Chain Monte Carlo (MCMC) parameters (chain length = 3,000,000, burn = 1,500,000, thin = 500) in MixSIAR with a residual error term (i.e., no process error). The model fit was evaluated with Gelmin-Rubin convergence diagnostics; i.e., R (Gelman et al. 2013), to ensure that all parameters returned R values <1.1.

**Table S1** δ^13^C values (‰) of α,ω-dicarboxylic fatty acids (diFAs) of surface soils under various land use and stream banks. Values within the row followed by the same superscript lower-case letter are not statistically significantly different at *p* < 0.05. Lower δ^13^C values indicate depletion of ^13^C in diFAs compared to international standards i.e., Vienna Pe De Belemnite (VPDB).

| Dicarboxylic fatty acid^a^ | Arable land | Pastureland | Woodland | Stream banks |
| --- | --- | --- | --- | --- |
| C_18_ | −34.8 ± 2.4^a^ | −34.5 ± 1.7^b^ | −32.4 ± 0.4^c^ | −32.7 ± 1.6^ab^ |
| C_20_ | −35.7 ± 2.1^a^ | −36.1 ± 1.8^b^ | −33.7 ± 1.2^c^ | −32.8 ± 0.9^ab^ |
| C_22_ | −37.0 ± 1.4^a^ | −37.7 ± 1.1^b^ | −34.7 ± 0.9^b^ | −34.1 ± 0.7^a^ |
| C_24_ | −37.0 ± 1.2^a^ | −37.0 ± 1.1^b^ | −34.4 ± 0.5^a^ | −35.0 ± 1.8^a^ |
| C_26_ | −37.3 ± 2.0^a^ | −36.5 ± 1.4^b^ | −33.9 ± 1.9^a^ | −35.2 ± 1.8^a^ |
| C_28_ | −33.0 ± 2.1 | −34.4 ± 2.6 | −34.1 ± 1.8 | −34.7 ± 2.4 |

^a^Subscript numbers indicate carbon numbers in the dicarboxylic fatty acids

**Figures**

**
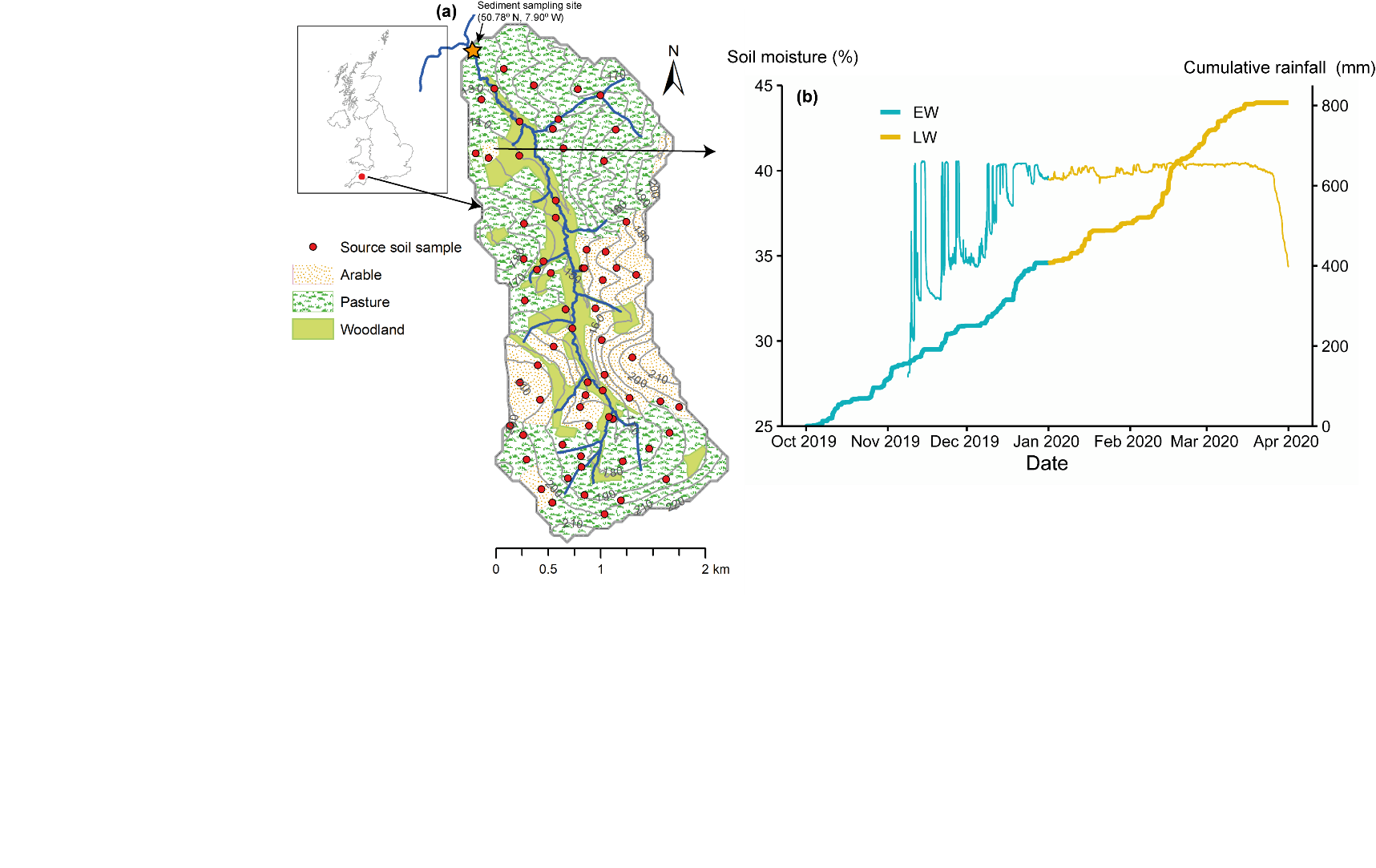
**

**Fig. S1** **a** Map of the Cocktree catchment showing the land use distribution and sediment source sampling points; **b** and the distribution of soil moisture at 10 cm depth (thin line) and cumulative rainfall (thick line) from arable land during the early winter (EW) and late winter (LW) periods (b). Sediments were collected at the catchment outlet during the winter of 2019–2020. The LW received about 6% more rainfall compared to EW at the time when the soil moisture was at the maximum level.


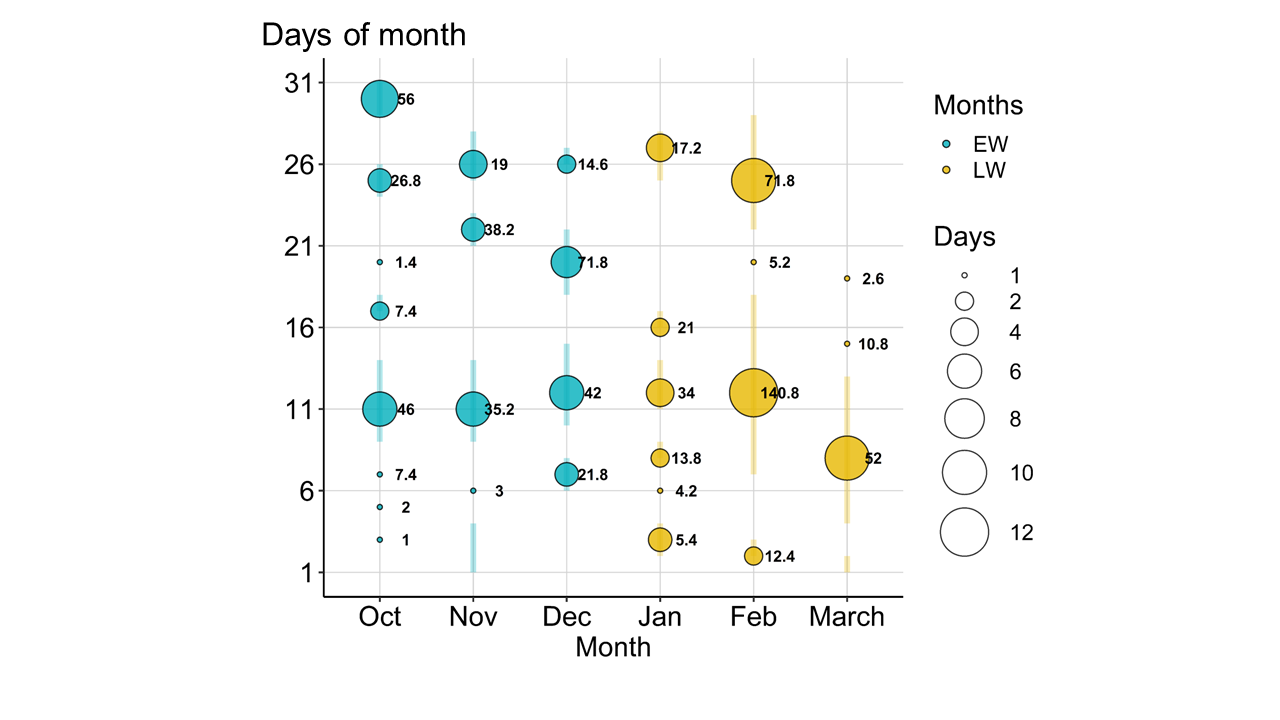


**Fig. S2** Bubble chart of the consecutive days with rainfall ≥ 1 mm in the early winter (EW) and late winter (LW) periods of 2019–2020. The number next to the bubble represents the total rainfall (mm) during the consecutive days. The rainfall distribution showed that LW has more consecutive days with rainfall ≥ 1 mm.


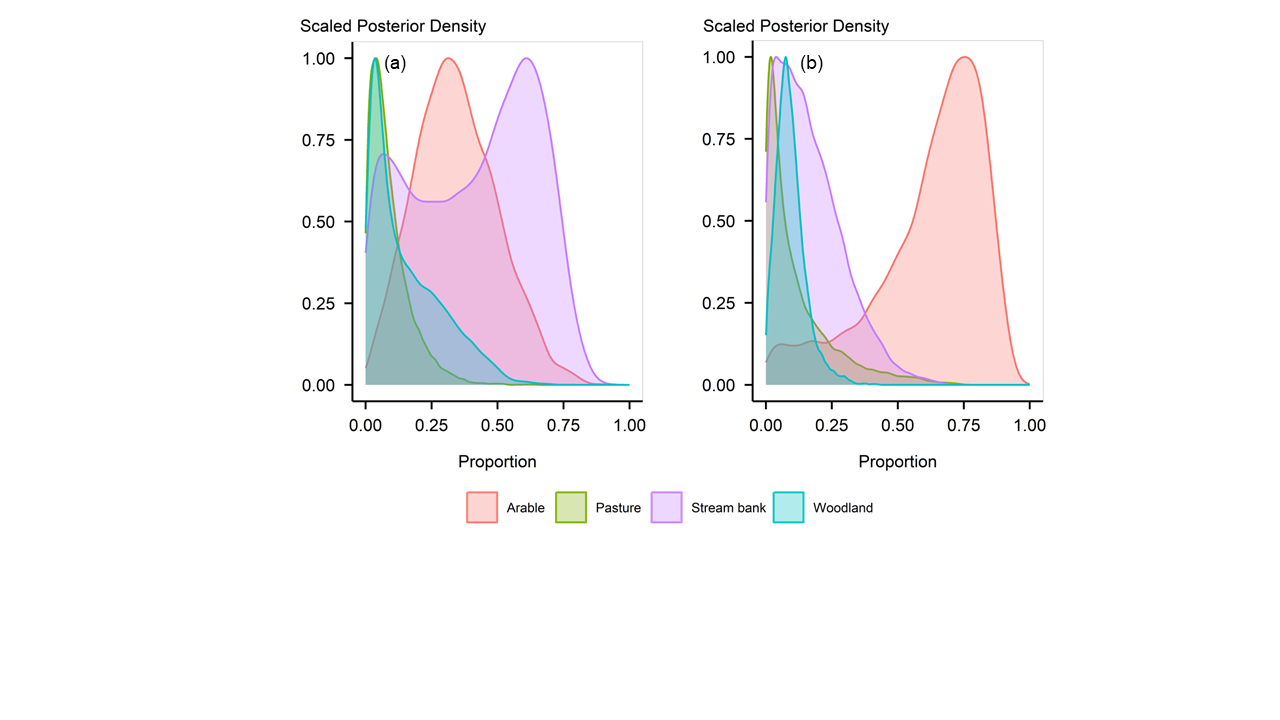


**Fig. S3** Estimated posterior distribution of source contributions to; **a** early winter; **b** late winter sediment using the concentration-dependent Bayesian mixing model (MixSIAR) without prior information.
